# Efficacy of Simparica and Simparica TRIO for the prevention of *Borrelia burgdorferi* by *Ixodes scapularis*

**DOI:** 10.1186/s13071-026-07328-x

**Published:** 2026-03-10

**Authors:** Thomas Geurden, Susan Holzmer, Jamie A. E. Myers, Kristina Kryda, Louise Longstaff, Riaan Maree, John W. McCall, Sean Mahabir, Jessica Rodriguez

**Affiliations:** 1 Zoetis, Veterinary Medicine Research and Development, Mercuriusstraat 20, 1930 Zaventem, Belgium; 2 Zoetis, Veterinary Medicine Research and Development, 333 Portage St., Kalamazoo, MI 49007 USA; 3 Zoetis UK Ltd., First Floor, Birchwood Building, Springfield Drive, Leatherhead, Surrey KT22 7LP UK; 4 Clinvet, 1479 Talmadge Hill Road South, Waverly, NY 14892 USA; 5 TRS Labs, Inc., 215 Paradise Boulevard, Athens, GA 30607 USA

**Keywords:** *Ixodes scapularis*, Lyme disease, *Borrelia burgdorferi*, Sarolaner, Prevention, Efficacy, Simparica, Simparica Trio

## Abstract

**Background:**

Both Simparica® and Simparica TRIO® chewable tablets are efficacious within 12 h against existing *Ixodes scapularis* infestations and within 24 h against re-infestations for 1 month. It is therefore expected that treatment with either product prevents Lyme infections due to their efficacy against *I. scapularis* ticks before the anticipated transmission of *Borrelia burgdorferi* by infected ticks*.*

**Methods:**

In total, four laboratory studies were conducted in which dogs were randomly allocated to two treatment groups of 10 dogs each. On day 0, dogs were either administered a placebo treatment (Pet-Tabs® Palatable Vitamin-Mineral Supplement for Dogs), Simparica TRIO tablets at the minimum dose of 1.2 mg/kg sarolaner, 24 μg/kg moxidectin and 5 mg/kg pyrantel (study 1 and 2) or Simparica at the minimum dose of 2 mg/kg sarolaner (study 3 and 4). On post-treatment day 28, each dog was infested with approximately 50 unfed, wild-caught adult *I. scapularis* ticks with a high *B. burgdorferi* infection rate. Blood samples were collected from each prior dog to treatment and on post-treatment days 27, 49, 63, 77, 91 and 104, and qualitatively tested for *B. burgdorferi* antibodies using the SNAP® 4Dx Plus and Lyme Quant C6® antibody tests. Four skin biopsies from each dog were collected on day 104 from the most common areas of tick attachment and tested by PCR for the quantitative presence of *B. burgdorferi.*

**Results:**

In all studies, at least nine out of 10 placebo-treated dogs were infected with *B. burgdorferi* before the end of the study. In study 1, one Simparica Trio-treated dog tested positive, whereas in the other studies none of the dogs treated with sarolaner tested positive at any time point during the study.

**Conclusions:**

Both Simparica and Simparica Trio at the minimum label dose prevent the transmission of *B. burgdorferi* infections as a direct result of killing the *I. scapularis* vector ticks.

**Graphical Abstract:**

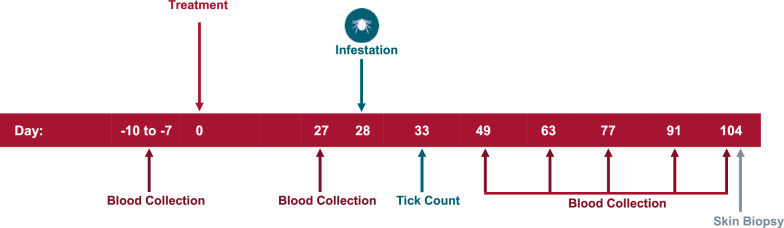

## Background

Ticks are obligate hematophagous ectoparasites of wild and domestic animals [1]. While around 40 *Ixodes* species have been identified in North America, *Ixodes scapularis* in the eastern states and *Ixodes pacificus* in the western states are most prevalent on domestic animals in the USA [2]. In a recent survey of ticks recovered by veterinarians, *I. scapularis* was present on 27.4% of infested dogs in the USA [3].

Lyme disease, caused by the spirochete bacterium *Borrelia burgdorferi*, is the most prevalent vector-borne illness in North America [4], posing a significant health threat to companion animals, particularly dogs, due to its potential to cause chronic symptoms ranging from fever and lethargy to severe joint, cardiac and neurological complications [5]. The pathogen is primarily transmitted by *I. scapularis* in the USA, apart from the Pacific coast region where it is mainly transmitted by *I. pacificus* [6]. Lyme transmission occurs when the tick injects the bacterial spirochete into the host during the bite event, usually within 24–48 h after the tick attaches to its host [7, 8]. Controlling exposure of dogs to ticks has therefore been an important prevention method to reduce the risk of *B. burgdorferi* infection, either by using acaricides or by the removal of ticks.

We report here the results of four studies aimed to evaluate the ability of Simparica® and Simparica TRIO® (Zoetis Inc., Parsippany, NJ, USA) chewable tablets, administered at their minimum doses, to prevent the transmission of *B. burgdorferi* infections from wild caught *I. scapularis* ticks by killing the ticks before transmission may occur. For both Simparica and Simparica Trio, the minimum label dose has previously been demonstrated to be effective within 12 h against existing *I. scapularis* infections and within 24 h against re-infestations for 1 month [9, 10]. It is therefore expected that the administration of either Simparica or Simparica TRIO will prevent the transmission of *B. burgdorferi* due to its efficacy in killing *I. scapularis* ticks before the likely transmission time has elapsed for *B. burgdorferi.*

## Methods

A total of four laboratory studies were conducted in accordance with the World Association for the Advancement of Veterinary Parasitology (WAAVP) guidelines for studies evaluating the efficacy of parasiticides in reducing the risk of vector-borne pathogen transmission in dogs and cats [7]. The study protocols were reviewed and approved by the Institutional Animal Care and Use Committee at each facility. All studies were placebo-controlled, masked and completely randomized. In study 1, the 20 Beagle dogs (15 males and 5 females) were between 10 and 11 months of age and weighed between 3.3 and 5.5 kg at the time of treatment. In study 2, the 20 Beagle dogs (9 males and 11 females) were between 8 and 9 months of age and weighed between 5.9 and 8.4 kg body weight at the time of treatment. In study 3, the 20 Beagle dogs (8 males and 12 females) were 9 months of age and weighed between 5.1 and 8.7 kg body weight at the time of treatment. In study 4, the 20 Beagle dogs (11 males and 9 females) were 11 months of age and weighed between 7.7 and 12.2 kg body weight at the time of treatment.

**Fig.1:**
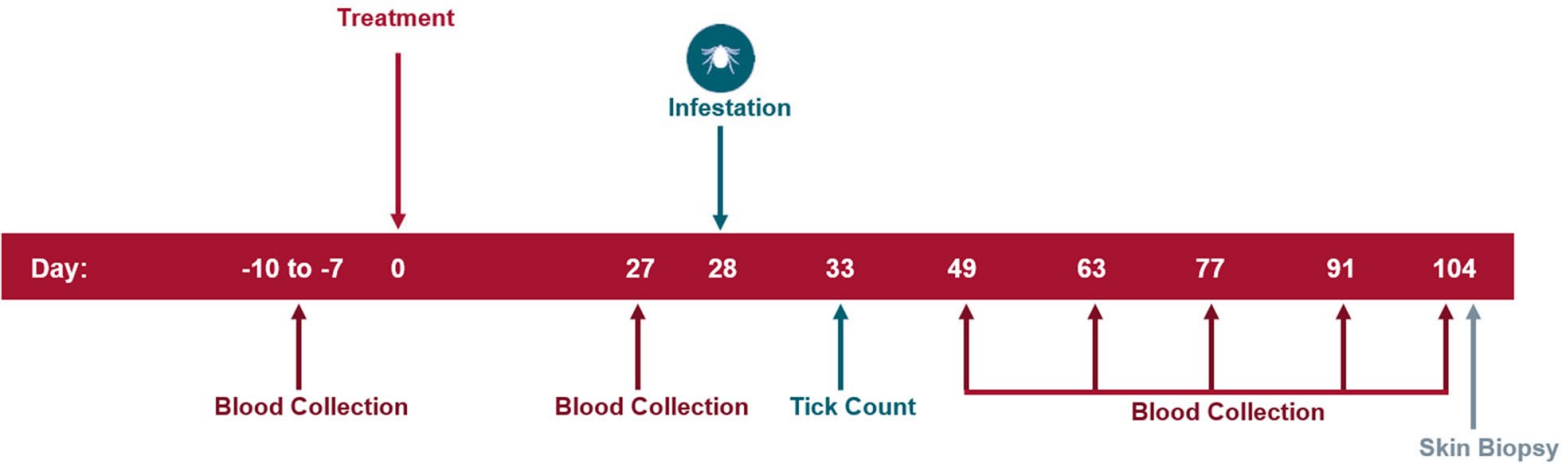
The study design for the induced infection studies with day of treatment (Day 0), day of tick infestation (Day 28) and tick counts (Day 33) and all sampling days

The study design is provided in Fig. 1. General health observations of the dogs were conducted at least once daily. In all studies, dogs were randomly allocated to two treatment groups. Nine dogs in the placebo group in study 1 finished the study; in each of the remaining groups across all studies 10 dogs finished the study. On day 0, one group was administered a placebo treatment (Pet-Tabs® Palatable Vitamin-Mineral Supplement for Dogs; Zoetis Inc.). The other group was administered Simparica TRIO tablets at the minimum dose of 1.2 mg/kg sarolaner, 24 μg/kg moxidectin and 5 mg/kg pyrantel (as pamoate salt) (studies 1 and 2) and Simparica at the minimum dose of 2.0 mg/kg sarolaner (studies 3 and 4). All treatments were administered orally. Whole tablets or combinations of tablets were administered so that dogs received the lowest possible dose without under-dosing. On day 28, each dog was infested with approximately 50 unfed, wild-caught adult *I. scapularis* ticks (approximately equal numbers of male and female ticks). The ticks in studies 1 and 3 were field-collected in Massachusetts, whereas those in studies 2 and 4 were field-collected in Rhode Island. Ticks were counted and removed on day 33. The viability of the ticks was assessed by gently blowing on them to expose them to CO_2_ or by applying tactile stimulus and observing any movement of the legs. Only live ticks (both attached and free-living ticks) were used in the efficacy calculations. The percent effectiveness of the treated group with respect to the placebo-treated group was calculated for day 33 as follows:

$${{\% Efficacy }} = \, \left( {\frac{{{\mathrm{Arithmetic}}\;{\text{ Mean }}\;{\text{Placebo }} - {\text{ Arithmetic }}\;{\text{Mean }}\;{\mathrm{Treated}}}}{{{\mathrm{Arithmetic}}\;{\text{ Mean}}\;{\text{ Placebo}}}}} \right) \times 100$$

Arithmetic means for live tick counts were estimated using the least squares means obtained from the statistical model. Tick counts were analyzed using a mixed linear model with treatment group as a fixed effect and error as a random effect. Treatment differences were assessed at the 5% level of significance.

The ticks in study 1 and 3 had a *B. burgdorferi* infection rate of 80% and 75%, respectively, whereas the ticks in study 2 and 4 had a *B. burgdorferi* infection rate of 75% and 60% respectively, as confirmed by PCR [10, 11]. Blood samples were collected from each dog pre-treatment (on days − 10 through − 7) and on post-treatment days 27, 49, 63, 77, 91 and 104 (see Fig. 1), and qualitatively tested for *B. burgdorferi* antibodies using the SNAP® 4Dx Plus Test (IDEXX Laboratories, Inc., Westbrook, ME, USA). Blood samples were also quantitatively assayed for *B. burgdorferi* antibodies using Lyme Quant C6® antibody tests (IDEXX Laboratories, Inc.). Four skin biopsies from each dog were collected on post-treatment day 104 from the heaviest areas of tick attachment or areas with the most notable inflammation from attached ticks, as recorded on day 33. Skin samples were tested by PCR for the quantitative presence of *B. burgdorferi* [12]. A dog was considered to be infected with *B. burgdorferi* as soon as one positive result was obtained after day 28 on the SNAP 4Dx Plus tests or the Lyme Quant C6 tests (titer ≥ 30 U/ml), or if any skin biopsy collected on day 104 tested positive by PCR. The proportion of animals infected (yes/no) with *B. burgdorferi* in the treated group was compared to the proportion of animals infected in the placebo group using Fisher’s exact test. The statistical test was performed at a significance level of 0.05 (two-sided).

## Results

No treatment-related adverse reactions were reported in any of the four studies. The placebo-treated dogs maintained adequate tick infestations on Day 33. The arithmetic mean tick count was 13.4 (range 4–20) in study 1, 23.4 (15–31) in study 2, 23.6 (15–31) in study 3 and 19.8 (15–28) in study 4. No ticks were observed on any of the treated dogs on Day 33 in studies 1, 2, and 3, leading to a 100% efficacy (*P* ≤ 0.0001) in these 3 studies. In study 4, two treated dogs were counted with 2–5 ticks and efficacy was 96.5% (*P* ≤ 0.0001).

The results for Simparica Trio tablets (studies 1 and 2) are provided in Table 1 and the results for Simparica tables (studies 3 and 4) are provided in Table 2. All dogs in these studies were seronegative for *B. burgdorferi* before treatment (pre-treatment and day 27), except for one placebo-treated dog in study 1 that was subsequently withdrawn from the study after a *B. burgdorferi*-positive Lyme Quant C6 test result for the blood sample collected on day 27, prior to infestation. In study 4, one placebo-treated dog remained negative for *B. burgdorferi* throughout the study. In studies 1, 2 and 3, all placebo-treated dogs were found to be positive for *B. burgdorferi* at all post-treatment days tested.

**Table 1:** showing the number of dogs testing positive for the different *Borrelia burgdorferi* assays in the placebo and treated group

Treatment	*Borelia burgdorferi* assays	Dogs positive for *B. burgdorferi*						
		Pre-treatment	Day 27	Day 49	Day 63	Day 77	Day 91	Day 104
*Study 1 (Massachusetts ticks, 80% infected with B. burgdorferi*)								
Placebo (*n* = 9)^a^	SNAP 4Dx Plus	0/9	0/9	1/9	9/9	9/9	9/9	9/9
	Lyme Quant C6	0/9	0/9	9/9	9/9	9/9	9/9	9/9
	Skin biopsy							9/9
Simparica Trio (*n* = 10)	SNAP 4Dx Plus	0/10	0/10	0/10	0/10	0/10	0/10	0/10
	Lyme Quant C6	0/10	0/10	1/10*	1/10^b^	0/10	1 of 10^b^	0/10
	Skin biopsy							1/10*
*Study 2 (Rhode Island ticks, 75% infected with B. burgdorferi)*								
Placebo (*n* = 10)	SNAP 4Dx Plus	0/10	0/10	1/10	7/10	10/10	9/10	10/10
	Lyme Quant C6	0/10	0/10	6/10	10/10	10/10	10/10	10/10
	Skin biopsy							10/10
Simparica Trio (*n* = 10)	SNAP 4Dx Plus	0/10	0/10	0/10	0/10	0/10	0/9^c^	0/10
	Lyme Quant C6	0/10	0/10	0/10	0/10	0/10	0/10	0/10
	Skin biopsy							0/10

^a^One placebo-treated dog was excluded from study after a *B. burgdorferi*-positive Lyme Quant C6 test result for the day 27 blood sample

^b^The same dog tested positive on subsequent days and tests

^c^One dog was excluded from the analysis due to a false-positive test result

**Table 2:** Results for Simparica (study 3 and 4), showing the number of dogs testing positive for the different *Borrelia Burgdorferi* assays in the placebo and treated group

Treatment	*Borelia burgdorferi* assays	Dogs positive for *B. burgdorferi*						
		Pre-treatment	Day 27	Day 49	Day 63	Day 77	Day 91	Day 104
*Study 3 (Massachusetts ticks, 75% infected with B. burgdorferi*)								
Placebo (*n* = 10)	SNAP 4Dx Plus	0/10	0/10	1/10	9/10	10/10	10/10	10/10
	Lyme Quant C6	0/10	0/10	6/10	10/10	10/10	10/10	10/10
	Skin Biopsy							10/10
Simparica (*n* = 10)	SNAP 4Dx Plus	0/10	0/10	0/10	0/10	0/10	0/10	0/10
	Lyme Quant C6	0/10	0/10	0/10	0/10	0/10	0/10	0/10
	Skin Biopsy							0/10
*Study 4 (Rhode Island ticks, 60% infected with B. burgdorferi)*								
Placebo (*n* = 10)	SNAP 4Dx Plus	0/10	0/10	0/10	9/10	9/10	9/10	9/10
	Lyme Quant C6	0/10	0/10	ND^a^	9/10	9/10	9/10	9/10
	Skin Biopsy							9/10
Simparica (*n* = 10)	SNAP 4Dx Plus	0/10	0/10	0/10	0/10	0/10	0/10	0/10
	Lyme Quant C6	0/10	0/10	ND^a^	0/10	0/10	0/10	0/10
	Skin Biopsy							0/10

^a^Samples collected on post-treatment day 49 were not examined using the Lyme Quant C6 test

In study 1, one Simparica TRIO-treated dog had three positive Lyme Quant C6 test results (titer ≥ 30 U/ml) and a low positive detection of *B. burgdorferi* via PCR for one of the four skin biopsies collected on day 104. The remaining nine Simparica TRIO-treated dogs (study 1) remained negative for *B. burgdorferi* throughout the study. In the three other studies, all dogs treated with Simparica or Simparica TRIO remained negative for *B. burgdorferi*, although in study 2 one treated dog had a false-positive (based on the other test results for that dog on day 91, and all other study days) SNAP 4Dx Plus test result on day 91. In these four studies, the proportion of dogs positive for *B. burgdorferi* in the treated groups was significantly (*P* ≤ 0.0001) lower than that in the placebo-treated group.

## Discussion

All but one of the placebo-treated dogs across the four studies tested positive for *B. burgdorferi*, demonstrating the validity of the infection model. The high infection (60–80%) rate of the ticks used for infestation and the minimum dose used for treatment as well as the timing of infestation allowed for evaluation of a worst-case scenario for potential *B. burgdorferi* transmission, and confirmation of efficacy for up to 28 days after treatment.

A previous study using the same model confirmed that the minimum dose of 2 mg/kg sarolaner prevents *B. burgdorferi* transmission by infected ticks [13]. These previous results are confirmed in studies 3 and 4, with a 100% prevention of transmission in both studies. While the dose of sarolaner is lower in Simparica Trio, it was previously demonstrated that the minimum label dose of 1.2 mg/kg sarolaner, 24 μg/kg moxidectin and 5 mg/kg pyrantel (as pamoate salt) resulted in a fast speed-of-kill, with efficacy within 8 h against existing *I. scapularis* infestations and within 24 h against re-infestations for 1 month [9]. The results of studies 1 and 2 confirm that the fast speed-of-kill of Simparica Trio leads to a reduction of the transmission of *B. burgdorferi* infection in at least 90% of the animals, as a direct result of killing *I. scapularis*.

To help prevent tick-borne diseases, pet owners are advised to minimize their pets’ contact with ticks, which can be achieved by the compliant use of acaricides. As the adult ticks of *I. scapularis* are most active and tend to feed on dogs during cooler months [14], it is crucial to ensure adequate tick prevention throughout the year in Lyme disease-endemic areas. Ticks generally need to be attached and feeding for 24–48 h before they can transmit *B. burgdorferi* infection [7, 8]. Eliminating infected ticks within this window can help prevent the spread of vector-borne diseases. Sarolaner, which acts rapidly and maintains high effectiveness within 24 h over 1 month following a single treatment, is well-suited to disrupt tick feeding and survival during this crucial period. Incorporating sarolaner (either Simparica or Simparica Trio) into a tick management strategy significantly lowers the risk of dogs contracting Lyme disease and reduces the harmful health impacts linked to tick infestations.

## Conclusions

The results of these four studies demonstrated that treatment with Simparica at the minimum dose of 2.0 mg/kg or Simparica Trio at the minimum dose of 1.2 mg/kg sarolaner, 24 μg/kg moxidectin and 5 mg/kg pyrantel (as pamoate salt) prevents *B. burgdorferi* infections as a direct result of killing the *I. scapularis* vector ticks.

## Data Availability

The data supporting the conclusions of this article are included in the tables within the article.
